# Interprofessional Perspective: Development of the National Academies of Practice Joint Health Literacy Position Statement

**DOI:** 10.3928/24748307-20260309-01

**Published:** 2026-04

**Authors:** Teresa Wagner, Vincent Salyers, Bich Nguyen, Tanya Sudia, Kate Taylor, Devora R. Winkfield, Nancy Colletti, Felicia Chew, Sarah Manspeaker

**Affiliations:** a College of Public Health, Community Health Worker Training Center, University of North Texas Health, Fort Worth, Texas;; b Joyce E. Lillis School of Nursing, Mercy College of Health Sciences, Des Moines, Iowa;; c Texas College of Osteopathic Medicine, University of North Texas Health Science Center, Fort Worth; d College of Nursing and Health Innovation, University of Texas at Arlington, Arlington; e College of Nursing, University of North Texas Health Science Center, Fort Worth, Texas;; f Howard University, Washington, DC;; g College of Medicine, University of Cincinnati, Cincinnati, Ohio;; h Thomas Jefferson University, Philadelphia and; i Athletic Training Program, Duquesne University, Pittsburgh, Pennsylvania.

The National Academies of Practice (NAP) Joint Health Literacy Position Statement (NAP, 2025), published in May 2025, addresses longstanding inconsistencies in how health literacy is defined, taught, and operationalized across health professions. This paper outlines the development of the position statement and the supporting evidence base. A structured review of existing health literacy frameworks across NAP's 17 professional academies was conducted, with each framework evaluated for use of common terminology (e.g., “health literacy,” “information literacy,” “communication accessibility”), emphasis on interprofessional collaboration (IPC), and integration of health equity principles. Findings revealed that only eight academies had formal frameworks, with significant variation in language, conceptual emphasis, and attention to equity (McGowan et al., 2020; American Society of Health-System Pharmacists [ASHP], 1997; Commission on Accreditation for Respiratory Care [CoARC], 2023). These inconsistencies hinder shared learning and collaborative care delivery, particularly for populations facing systemic barriers (American Public Health Association [APHA], 2014; American Physical Therapy Association [APTA], 2019). Drawing on best practices and cross-cutting gaps identified during the review, the Joint Health Literacy Working Group synthesized a unified, interprofessional framework that explicitly integrates health equity and outlines shared responsibilities across disciplines. The resulting position statement provides a roadmap for advancing coordinated, person-centered communication and care. Its implementation has the potential to reduce health disparities, strengthen interprofessional education, and align practice standards with national equity goals (Office of Disease Prevention and Health Promotion, n.d.;)World Health Organization [WHO], 2010).

“Health literacy is a multifaceted concept encompassing personal, interpersonal, organizational, navigational, functional, numeracy, and digital dimensions, each influencing an individual's ability to access, understand, and use health information effectively” (Ratzan & Parker, 2000; WHO, 2010). Health literacy frameworks across various health professions share a common goal of enhancing individuals' capacity to make informed decisions about their own health and the health of others (Academy of Medical-Surgical Nurses [AMSN], n.d.; American Speech-Language-Hearing Association [ASHA], n.d.). However, the absence of a standardized, interprofessional approach has hindered collaborative efforts to improve health literacy and address related disparities (CoARC, 2023; Madden & Tupper, 2024).

In Fall 2024, the NAP Interprofessional Collaboration Committee convened a Joint Health Literacy Working Group (JHLWG) to develop an interprofessional health literacy position statement aligned with Healthy People 2030 (Office of Disease Prevention and Health Promotion, n.d.) goals and its leading health indicators objectives. As the JHLWG began its work, it discovered that only eight of the seventeen Academies within NAP had formal health literacy frameworks, and those that existed varied considerably in terminology, emphasis on interprofessional collaboration, and integration of health equity principles (NAP, 2025). The group identified a critical need to address these gaps. Inconsistent terminology and fragmented frameworks made it difficult to develop shared goals, communicate effectively across professions, and coordinate already complex interventions. A unified approach was needed to support integrated, equitable, and effective care delivery.

## Methods

“We conducted a literature scan of academic databases, gray literature, and professional association publications across the seventeen health disciplines represented in NAP, aiming to identify both well-established and emerging health literacy models.” Each framework was evaluated according to three foundational tenets: (1) use of common terminology, specifically the use of “health literacy” or equivalents such as “information literacy” or “communication accessibility,” (2) emphasis on IPC, and (3) integration of health equity principles. Frameworks were analyzed for the explicitness and centrality of these tenets. A fourth category, alternative terms, was added because a framework may have used terms outside of the three foundational ones. **Table 1** provides a comparative analysis of these terms used to identify cross-cutting gaps, inconsistencies, and best practices, which ultimately shaped the unified approach presented in the NAP position statement.

**Table 1:**
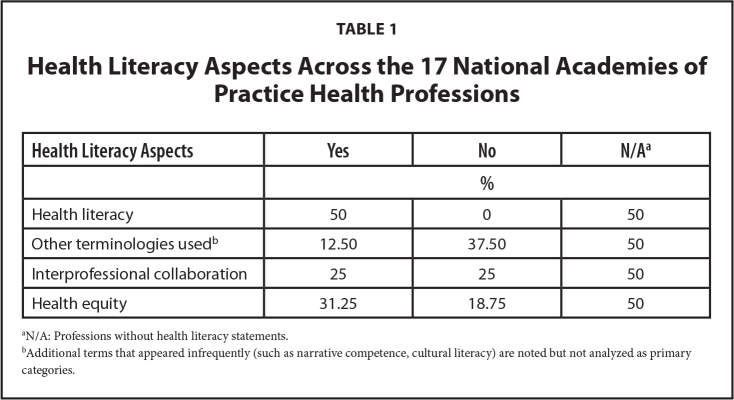
Health Literacy Aspects Across the 17 National Academies of Practice Health Professions

**Health Literacy Aspects**	**Yes**	**No**	**N/A^a^**
	**%**		
Health literacy	50	0	50
Other terminologies used^b^	12.50	37.50	50
Interprofessional collaboration	25	25	50
Health equity	31.25	18.75	50

a N/A: Professions without health literacy statements.

b Additional terms that appeared infrequently (such as narrative competence, cultural literacy) are noted but not analyzed as primary categories.

## Results

### Common Terminology

Health literacy frameworks generally define the concept as the capacity of individuals to obtain, process, and understand basic health information and services needed to make appropriate health decisions (Ratzan & Parker, 2000; WHO, 2010; WHO, 2021). Despite agreement on this foundational definition, terminology diverges significantly across professions, shaped by their distinct educational and practice priorities. In nursing, for example, the term “information literacy” is often emphasized in academic settings, focusing on skills like database use and research interpretation. McGowan et al. (2020) found that nursing faculty frequently emphasize academic research over real-world applications such as patient navigation, understanding discharge instructions, or medication adherence.

In the fields of audiology and speech-language pathology, health literacy is often tied to communication accessibility. The American Speech-Language-Hearing Association (ASHA, n.d.) emphasizes health literacy as a part of patient-centered communication but tends to interlink it with communication disorders. In contrast, public health professionals use a more expansive interpretation of health literacy to address systemic disparities and advocate for structural interventions. For instance, the APHA (2010) highlights health literacy in the context of federal coordination and community-based engagement.

These varying definitions, ranging from information literacy to cultural competence and patient education, can result in semantic fragmentation. A physician may view health literacy through the lens of patient compliance, while a nurse sees it as an educational task, and a speech-language pathologist may focus on cognitive and linguistic barriers. Without shared language, patient education and communication strategies can become disjointed, particularly during care transitions.

These semantic differences not only impede interdisciplinary alignment but also act as tangible barriers to coordinated, interprofessional education and care. Without being taught shared language, health teams may struggle to build cohesive communication strategies, particularly during care transitions or when working with vulnerable populations.” Adopting a unified language would enhance interprofessional education and better prepare future practitioners for coordinated patient-centered care.

### Interprofessional Collaboration

Many professional frameworks promote IPC as essential to enhancing health literacy. The WHO's (2010) Framework for Action on Interprofessional Education and Collaborative Practice calls for professions to collaborate in delivering care that meets population health needs, emphasizing shared communication and decision-making. However, implementation remains inconsistent.

Despite the WHO's framework, many professional bodies address IPC inconsistently or in isolation. For example:

The APTA (2019) positions health literacy as foundational to achieving a “just and inclusive society,” linking it to systemic equity. However, their framework does not detail how physical therapists should engage with nurses, pharmacists, or dietitians to coordinate patient education.

The American Society of Health-System Pharmacists (ASHP, 1997) underscores the role of pharmacists in counseling and medication management but tends to view collaboration through a pharmacy practice lens rather than integrated care teams.

The CoARC (2023) includes interprofessional communication as an accreditation standard, indirectly promoting IPC, yet leaves implementation strategies largely undefined.

Barriers to IPC include differences in educational training, varied emphasis on patient communication, and discipline-specific priorities. For instance, occupational therapists may focus on modifying environments to support functional living, while dietitians often emphasize behavior change through nutrition literacy, despite lacking a formal framework for health literacy. Without a unifying model, these efforts remain siloed. A notable exception is athletic training, where Madden and Tupper (2024) advocate for embedding “Health Literacy Champions” within care teams, exemplifying how interdisciplinary leadership can emerge from less traditional clinical roles.

Fragmented frameworks not only affect professional standards but also limit students' exposure to interprofessional collaboration by reducing opportunities to build shared competencies in health literacy. Integrating a consistent framework into academic settings can bridge this educational gap and foster more collaborative, patient-centered care across disciplines.

### Health Equity Integration

Health literacy is fundamentally connected to health equity and the mitigation of health disparities (APHA, 2010; APTA, 2019). Individuals with limited health literacy face increased risks of mismanaging chronic conditions, medication misuse, hospital readmissions, and poor adherence to treatment plans (ASHP, 1997; McGowan et al., 2020). These challenges are magnified among groups that encounter systemic barriers, including people with disabilities, limited English proficiency, lower educational attainment, and members of historically marginalized racial or ethnic communities (AMSN, n.d.; Milken Institute, n.d.; NAP, 2025).

Yet the degree to which health equity is integrated into health literacy frameworks varies. APTA (2019) explicitly frames health literacy as a pathway to social justice, advocating for dismantling systemic inequities alongside individual education efforts. APHA (2010) connects low health literacy to structural issues and promotes policy interventions, such as reforming educational systems and tailoring outreach for underserved populations. In contrast, the American Student Dental Association (as cited in APHA, 2010) calls for including health literacy in dental curricula but does not directly connect it to equity or disparities, despite persistent gaps in oral health by race and income.

Nursing frameworks (AMSN, n.d.) typically emphasize patient-centered care and advocacy but may insufficiently address root causes of limited health literacy—such as poverty, racism, or educational inequity. Respiratory care frameworks, particularly in asthma education, implicitly address equity by acknowledging the psychosocial and socioeconomic variables influencing patient care, though without a formalized equity lens. This inconsistent treatment of health equity hinders the development of a shared, public-health-oriented framework for health literacy across professions.

Educationally, this lack of integration leaves students underprepared to recognize and address structural barriers that affect patient comprehension and engagement. It limits opportunities to develop the critical reflection skills needed to navigate power dynamics, cultural contexts, and social determinants of health, ultimately undermining their ability to deliver equitable, person-centered communication as part of interprofessional teams.

## Discussion

The NAP Joint Health Literacy Position Statement addresses disciplinary discrepancies by establishing a common language, explicitly promoting interprofessional collaboration, and embedding health equity as a foundational principle. It unifies terminology across disciplines, outlines shared professional responsibilities and provides actionable guidance to integrate health literacy into practice, education, and policy (NAP, 2025). This coordinated framework supports more equitable, person-centered care by ensuring that health literacy efforts are consistent, inclusive, and aligned with the real-world needs of diverse populations (Coleman, 2013).

Incorporation of a unified health literacy framework is essential for optimal care outcomes. Current operational fragmentation, including differences in terminology, collaboration, and equity integration across health professions, hinders coordinated, patient-centered approaches. Advancing health literacy as a cross-cutting priority will require action in these areas:

The development of standardized frameworks that incorporate a shared vocabulary, explicitly link health literacy to health equity, and outline interprofessional roles (NAP, 2025; WHO, 2010; WHO, 2021).

The inclusion of health profession curricula that includes interdisciplinary training in patient communication, shared decision-making, and cultural humility (AMSN, n.d.; ASHA, n.d.; McGowan et al., 2020; Nutbeam, 2000).

Policymakers and accrediting bodies must incentivize integrated approaches to health literacy as part of care quality and equity metrics

(APHA, 2010; ASHP, 1997; CoARC, 2023).

The statement explicitly embeds health equity as a core component of health care, ensuring that health literacy efforts are inclusive and responsive to the needs of diverse and marginalized populations. By doing so, the statement fosters more coordinated, equitable, and person-centered care (Coleman, 2013) across the health system and articulates a shared understanding of health literacy, providing actionable recommendations for its integration into education, practice, and policy.

Creating a more unified, inclusive vision of health literacy requires an interprofessional approach to both structural reforms and grassroots leadership from each profession. The National Academies of Practice Joint Health Literacy Position Statement (NAP, 2025) represents a significant leading step toward this goal by facilitating collaborative efforts to enhance health literacy and address disparities. Continued commitment to collaboration and equity is essential. The development of this joint health literacy statement also serves as a model for broader interprofessional collaboration. It illustrates how shared goals can bridge professional silos and advance person-centered care. Ultimately, embedding an IPC ethos into organizational culture is essential, not only for improving health literacy but also for transforming health systems to better meet the needs of patients and communities.
